# Does wearing facial masks increase perceived facial attractiveness? An eye-tracking experiment

**DOI:** 10.3389/fpsyg.2023.1141319

**Published:** 2023-05-12

**Authors:** Nan-Hee Jeong, Junsik Lee, Ji-Chan Yun, Do-Hyung Park, Se-Bum Park

**Affiliations:** ^1^School of Business, Yonsei University, Seoul, Republic of Korea; ^2^Graduate School of Business IT, Kookmin University, Seoul, Republic of Korea

**Keywords:** mask, mask-fishing, covering effect, facial attractiveness, eye-tracking, COVID-19

## Abstract

As wearing a mask has become a routine of daily life since COVID-19, there is a growing need for psycho-physiological research to examine whether and how mask-fishing effects can occur and operate. Building upon a notion that people are likely to utilize information available from the facial areas uncovered by a mask to form the first impression about others, we posit a curvilinear relationship between the amount of the facial areas covered by a mask and the perception of others’ attractiveness such that the attractiveness perception increases initially and then decreases as more facial areas are covered by a mask. To better examine this covering effect, we conduct an experiment using an eye-tracker and also administer a follow-up survey on the facial attractiveness of target persons. Our results showed that the facial attractiveness of target persons increased as the areas covered by a mask increased as in the moderate covering condition where the target persons wore only a facial mask, demonstrating that the mask-fishing was indeed possible thanks to the covering effect of a mask on the facial attractiveness. The experimental results, however, revealed that the mask-fishing effect disappeared as the areas covered increased further as in the excessive covering condition where the target persons’ face and forehead were covered with a mask and a bucket hat. More importantly, the eye-tracking data analysis demonstrated that both the number of gaze fixation and revisits per unit area were significantly lower in the moderate covering than in the excessive covering condition, suggesting that participants in the moderate covering were able to form the impression about the target persons using cues available from the eyes and forehead areas such as hairstyle and eye color whereas those in the excessive covering were provided only a limited set of cues concentrated in the eyes area. As a result, the covering effect no longer existed under the excessive covering. Furthermore, our results showed that participants in the moderate covering were more likely than those in the excessive condition to exhibit the higher level of curiosity and perception of beautifulness but perceived the lower level of coldness when evaluating the target persons. The current research offers theoretical contributions and practical implications made from the eye-tracking experiment and discusses possible avenues for further research.

## 1. Introduction

The outbreak of COVID-19 in 2019 has brought a lot of significant changes to daily life at the individual, social, and national level. One of the most significant changes is the mandatory wearing of masks (Rab et al., 2020). According to the World Health Organization (WHO) recommendation in January 2020, the mandatory wearing of masks has begun to protect ourselves and others from infectious diseases, but as a result, the life of covering part of our true selves also began. A mask covering almost half of the face often felt stuffy and uncomfortable when communicating with others especially in hot weather (Ribeiro et al., 2020; Green et al., 2021), and also made it difficult to grasp the other person’s emotions or intentions (Carbon, 2020; Leitner et al., 2022; Parada-Fernández et al., 2022). Nevertheless, most people manage to live with the hassle of wearing a mask every day due to the medical benefits in the prolonged pandemic era.

As each country has begun to loosen the quarantine standards and regulations with growing expectations for the end of COVID-19, wearing masks is no longer a requirement or obligation. Surprisingly, however, it is not at all difficult to find people still insisting on wearing masks. According to a survey of 1,217 Korean adults, 80% of the respondents said that they would continue to wear a mask even if COVID-19 ended (Park, 2022). According to a recent article in *The New York Times*, for example, some people in South Korea and Japan still wear masks because the everyday burden of makeup and facial expression management is likely to be reduced (John and Hida, 2023). While hygienic factors such as self-quarantine may account for the continued use of masks (Burgess and Horii, 2012), this continued need for wearing a mask may also be explained by a newly coined term called “mask-fishing.” Mask-fishing, which is a combination of the words “mask” and “fishing,” is referred to as a phenomenon where people look surprisingly more attractive when wearing a mask than when not wearing one. We assume that this mask-fishing effect would be one plausible reason most of the survey respondents in Korea are afraid of taking off masks. Hies and Lewis (2022), for example, demonstrated that wearing a mask led to positive person perceptions such that women rated men wearing a blue medical mask as most attractive, followed by those wearing a white cloth mask relative to those without a mask or those covering a part of their face with a black book because wearing a mask, especially one in blue color, reminded them of medical staffs. Relatedly, Dudarev et al. (2022a) found that individuals wearing a mask were perceived as more attractive than those not wearing a mask as long as they were of the same race whereas masked individuals of another race were considered less attractive relative to those unmasked. Also, Dudarev et al. (2022b) found that both the frequency and the duration of wearing a protective mask were positively associated with the emotional appraisal of it, suggesting that familiarity with the protective mask would breed the liking of it. According to recent studies, however, individuals experienced a greater level of difficulty grasping other people’s facial expressions when they were wearing a mask than when not wearing one (Koster et al., 2007; Dhamecha et al., 2014; Carbon, 2020). Leitner et al. (2022) also found that individuals experienced greater uncertainty and lesser intensity recognizing basic emotions from the facial expressions of people who were masked than unmasked. In a similar vein, Parada-Fernández et al. (2022) showed that wearing a mask positively affected the attractiveness of facial expressions in both genders with regard to emotions such as sadness, surprise, and anger except for happiness despite the shortcomings of recognizing emotions such as happiness, sadness, and anger. In addition, Miyazaki and Kawahara (2016) maintained that wearing a sanitary mask decreased the perception of facial attractiveness as the mask primed unhealthiness and evoked more thoughts about diseases, resulting in a desire to avoid masked individuals.

Despite all the pros and cons of wearing a mask as mentioned above, wearing a mask has become a routine of daily life since COVID-19. Given this seeming inconsistency, there arises a growing need for psycho-physiological research to investigate whether or not wearing a mask would lead to a positive or negative person perception, and how such covering effect of wearing a mask would operate in a psycho-physiological manner. The results of recent studies in the following may provide a basis for an important underlying process in addressing the issues just raised. Kamatani et al. (2021), for example, found that the faces of individuals considered less (more) attractive were likely to be perceived as more (less) attractive with a mask than without a mask because wearing a mask took the viewers’ attention away from the areas covered by the mask and relocated it to those uncovered by the mask. Relatedly, Leitner et al. (2022) demonstrated that social or fake smiles with a mask were rated as more honest and genuine than those without a mask perhaps because wearing a mask made it difficult to observe the muscles around the mouth and thus to process information with regard to such smiles in a complete manner.

On the other hand, prior research on curiosity has shown that upon exposure to new stimuli or objects such as a new gift most individuals were naturally motivated to explore and learn more about these targets to satisfy their curiosity, often resulting in positive evaluations of the target objects (Berlyne, 1949; Loewenstein, 1994; Litman and Spielberger, 2003). Kashdan et al. (2011), for example, found that greater curiosity increased the level of intimacy and contributed to more positive social interactions when socializing with an unacquainted stranger. In a similar vein, a recent study by Rixom et al. (2020) indicated that giving a gift wrapped in pretty wrapping paper increased the level of expectation and curiosity a gift recipient had about the gift and evoked positive emotions as a result, compared with giving the same gift in an open box. Likewise, exposing some of a new product or making it transparent not only provided minimal visual cues necessary to making an informed judgment about the new product, but also prevented a buyer from having an excessive expectation that often led to negative post-purchase dissatisfaction (Hagtvedt and Patrick, 2008; Patrick et al., 2017). However, individuals exposed to completely new stimuli or given the limited amount of stimulation began to experience physiologically unpleasant states and ultimately exhibited avoidance behavior by refusing to explore the targets further (Hebb, 1955; Leuba, 1955).

Building upon this cue utilization theorizing, we hypothesize that wearing a mask has also a covering effect such that wearing a mask can stimulate a viewer’s curiosity about a target person by covering part of all of his or her face, and the induced curiosity can in turn relocate to increasing interest and eye gaze fixation to the unmasked areas, such as the eyes and forehead, from which all the available cues with regard to the target perception are inferred. Unfortunately, this covering effect that wearing a mask has on positive target perceptions, however, may have an upper limit. That is, covering more facial areas of a target with a hat or sunglasses may not always lead to a more positive target perception as cues become extremely limited, which in turn reduces a viewer’s curiosity and interests about a target person. When making sense of one covering too much of the face with a mask, a hat, and sunglasses, people are more likely to generate negative thoughts rather than express curiosity about him or her. Thus, we expect a curvilinear relationship between the amount of the facial areas covered by a mask and the perception of attractiveness of mask wearers, such that the attractiveness perception would increase initially and then decrease as more facial areas are covered by a mask.

To test our hypotheses, the current research conducts an eye-tracker experiment and a follow-up self-administered survey in which research participants view the images of people wearing a mask with the varying degrees of facial covering, and rate then the facial attractiveness of target persons in a follow-up survey. Further details on our eye-tracking experiment and the survey are provided in the following.

## 2. Methodology

To investigate the effect of wearing a mask on the facial attractiveness and the psycho-physiological underpinnings of the mask’s covering effect, the current research employed an eye-tracking method in order to capture and measure human eye movements and eye gaze patterns in almost real-time (Isaacowitz et al., 2006). An eye-tracker used in this study is the GP3 model released by Gazepoint that records the data measured at 60 Hz per second and its gaze tracking tool measures a participant’s reaction by recognizing his or her pupil shape through infrared rays. The areas of interest (AOI) in the visual stimulus of a mask wearer are set and the fixation and the revisit score for each AOI are estimated. The fixation score is calculated as one time when the pupil is fixed for 0.05 s or more without moving so that understanding of the places people focus on in evaluating a target object through fixation is made possible. The revisit score is calculated as the number of times the eye gaze is off the field of interest and turning back. Through the gaze movement between the AOIs we aim to identify the area of reference when participants are viewed the image of target persons wearing a mask to a varying degree of facial covering.

### 2.1. Design and participants

The current research employed a one-factor within-subject experimental design in which the covering level was manipulated as the following four conditions: a moderate covering, an excessive covering, an uncovered baseline to the moderate covering, an uncovered baseline to the excessive covering. While a target person, either a male or a female, in the moderate covering condition was wearing a mask, the identical person was wearing both a mask and a bucket hat in the excessive covering condition. Each of the two uncovered baseline conditions in which a target male or female wore neither a mask nor a bucket hat served as a no treatment control group to the moderate and the excessive covering condition, respectively, when it comes to comparing facial attractiveness. Despite the strict restriction that participants were required to visit our laboratory and to wear an eye-tracker during about a 40-min-long experiment, 26 undergrads recruited from a private university in South Korea volunteered to participate in the experiment for extra course credit. Of the 26 participants, one participant who did not respond to the questions to the end was excluded and the data from the 25 participants were used for subsequent analysis ( $M_{age}$ =22.60, 60% female).

### 2.2. Stimuli material

The original photos of a male and a female used in our stimuli were obtained from an online database and were modified for only academic purpose. We selected the photos of Korean male and female in their twenties to control the effect of race and age on person perception. As a result, the upper-body photos of a male and a female target whose faces were observable were used to manipulate the following four covering levels. The lower part of the target person’s face, the area corresponding to his or her mouth and chin, was covered with a mask in the moderate covering condition. By contrast, in the excessive covering condition, the lower part of the target person’s face and the upper part of the target person’s eyes (i.e., the target’s forehead) were covered with a mask and a bucket hat, respectively. The rest of the photos represented the uncovered baseline conditions to the moderate and the excessive covering condition, respectively. All stimuli photos were presented in the same 428 × 296 pixels size and other confounding factors (i.e., hairstyles, background etc.) were eliminated as much as possible.

### 2.3. Procedures

**Figure 1:**
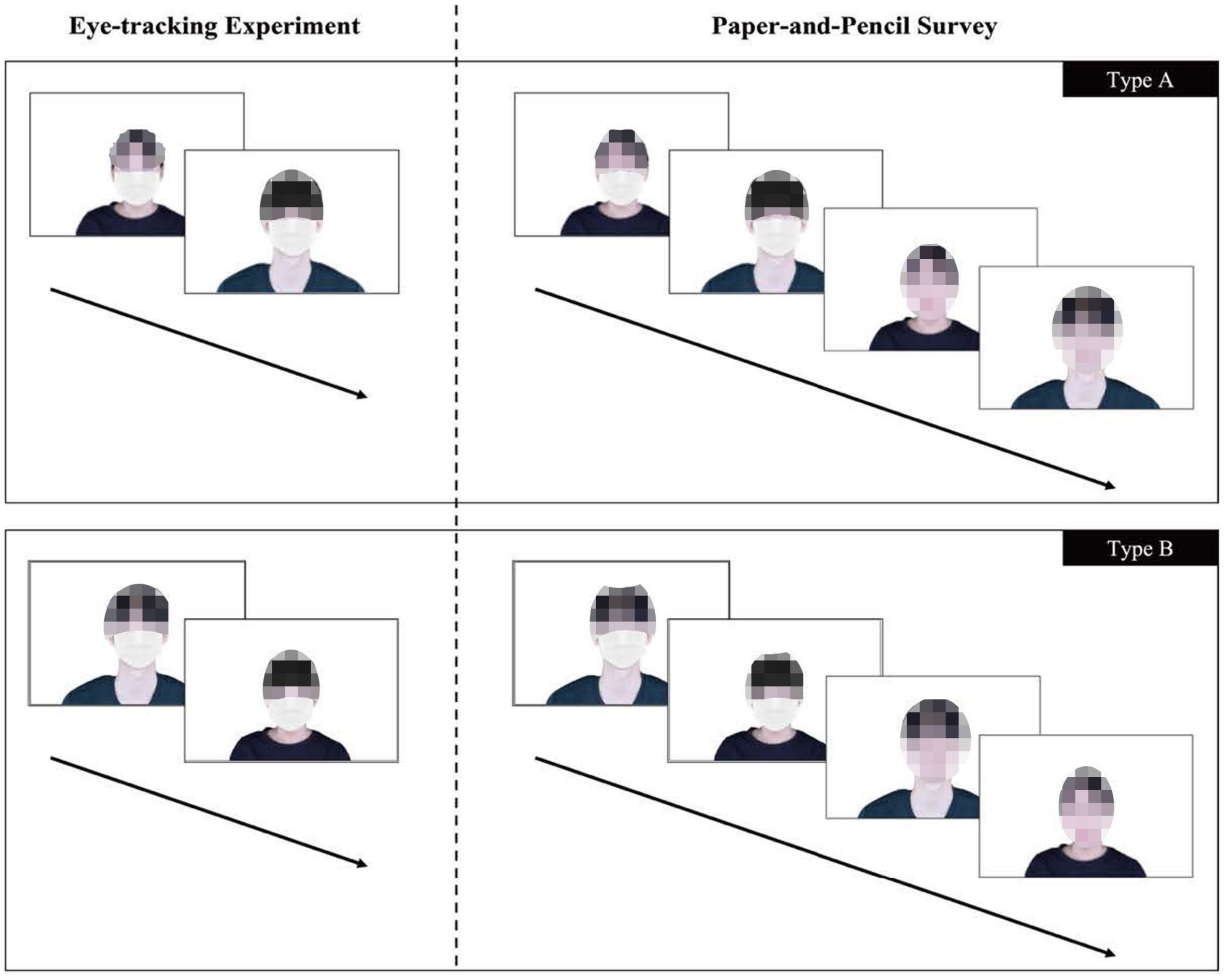
Experiment stimuli and procedures: the photos above are, from upper-left to lower-right, the moderate covering (a mask only), the excessive covering (a mask and a bucket hat) in the eye-tracking experiment. With two covering conditions, the uncovered baseline conditions to the moderate and the excessive covering condition, respectively, were added in the paper-and-pencil survey. All participants were randomly assigned to type A or B. Facial images reproduced with permission from AI-Hub (https://www.aihub.or.kr), operated by the National Information Society Agency in Korea.

As shown in Figure 1, the current experiment was run in two parts in which each participant’s gaze data were gathered through the eye-tracker and each participant’s perception about the target persons was measured by a paper-and-pencil self-administered survey. Upon arrival at the laboratory each participant was instructed to fill out a written consent form. Next, each participant was asked to wear the eye-tracker equipment and to fix their gaze on a specific point. Afterward, each participant looked at the stimuli photos through a monitor under the time restriction set to 15 s. Our participants observed a total of two photos in which the covering level was manipulated as either a moderate covering or an excessive covering condition. In so doing, we alternately presented the gender of the two target persons such that if a participant was shown a photo of a female (male) target wearing a mask in the moderate covering condition, he or she was presented with a photo of a male (female) target wearing a mask and a bucket hat in the excessive covering condition. By alternating the gender of the two target persons, we managed to minimize a possible gender bias. After the eye-tracker experiment, participants were asked to move to a nearby seat and to complete a paper-and-pencil self-administered survey on their perceptions of the target persons.

Next, participants who competed the eye-tracker task were shown once again the same set of the photos as in the eye-tracker experiment in the paper-and-pencil self-administered survey. In the survey, each participant completed the following two 7-point Likert items (1 = not at all; 7 = very much) that were used to measure the facial attractiveness of the target persons shown in the moderate and the excessive covering condition: “I like the target person in the picture,” “The target person in the picture is attractive.” Across the four experimental conditions these items were averaged to form a reliable facial attractiveness index (*𝑟* = 0.46, *p* < 0.01). Also, participants were asked to complete another two 7-point Likert items (1 = not at all; 7 = very much) that were used to measure curiosity participants had about the target persons shown in the moderate and the excessive covering condition: “I am curious about the target person,” “I want to see the target person’s face.” Again, these two items were averaged to form a reliable curiosity index (*𝑟* = 0.37, *p* < 0.01). Next, the following bipolar scales were used to measure participants’ perception of coldness in relation to their first impression of the target persons (1 = warm; 7 = cold) and subjective perception of beautifulness (1 = ugly; 7 = beautiful) shown in the moderate and the excessive covering condition. Afterward, participants rated the attractiveness of the two target persons as in the uncovered baseline condition such that participants were asked to measure the same facial attractiveness items (liking, attractiveness) with respect to a female (male) without a mask and a male (female) without a mask and a bucket hat, respectively. Last, participants were asked to leave after reporting their age and gender.

### 2.4. Statistical analysis

We used IBM SPSS 25.0 to analyze the data from the eye-tracking experiment and the paper-and-pencil survey. A paired *t*-test was mainly used as a statistical method as the current research employed a one-factor within-subject experimental design.

## 3. Results

### 3.1. The effect of wearing a mask on the perception of facial attractiveness

**Figure 2:**
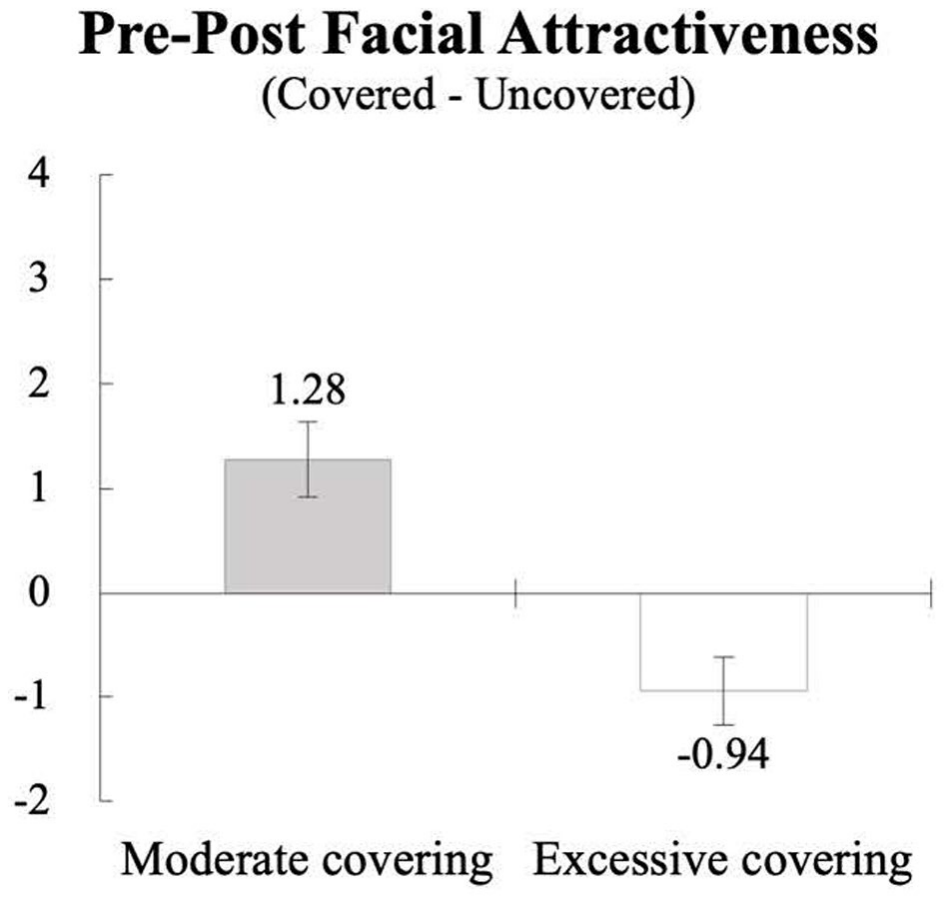
The effect of wearing a mask on the facial attractiveness index. The numbers are the mean difference between the covered (pre) and the uncovered baseline (post). The error has show ± one standard error calculated from within participant variance.

First, a paired *t*-test on the facial attractiveness index was performed to test whether wearing a mask would increase or decrease a viewer’s perception of the facial attractiveness of those mask wearers. As shown in Table 1 and Figure 2, the analysis revealed that participants perceived the target persons under the moderate covering (*M* = 4.34, SD = 1.74) to be more attractive than the same persons under the uncovering baseline (*M* = 3.06, SD = 1.32; *t*(24) = 3.54, *p* < 0.01), suggesting that wearing a mask led to a statistically significant increase in the facial attractiveness index. The reverse was true, however, in the excessive covering condition: the targets wearing both a mask and a bucket hat under the excessive covering (*M* = 2.66, SD = 1.01) were rated less attractive than the same targets under the uncovering baseline (*M* = 3.60, SD = 1.41; *t*(24) = −2.81, *p* < 0.01), suggesting that covering too much of the face backfired the perception of facial attractiveness. Because the mean difference between the baseline conditions was not statistically significant (*t*(24) = −1.84, *NS*), the average facial attractiveness index of the two baseline conditions was used for further analysis to test the curvilinear effect of wearing a mask.

**Table 1:** Means and standard deviations (in parentheses) across the experimental conditions.

	Moderate covering (only a mask) (A)	Excessive covering (a mask with a bucket hat) (B)	Uncovered baseline		*t*-Statistic (two-tailed) (A)–(B)
			To the moderate covering	To the excessive covering	
Facial attractiveness index	4.34 (1.74)	2.66 (1.01)	3.06 (1.32)	3.60 (1.41)	*t*(24) = 5.38***
Curiosity index	3.70 (1.80)	3.24 (1.44)	---	---	*t*(24) = 1.79†
Beautifulness	4.76 (1.27)	4.08 (0.49)	---	---	*t*(24) = 2.59*
Coldness	4.84 (1.21)	6.24 (1.42)	---	---	*t*(24) = −4.67***

^†^*p* < 0.10, * *p* < 0.05, ** *p* < 0.01, *** *p* < 0.001.

Second, the same analysis on the curiosity index indicated that participants under the moderate covering (*M* = 3.70, SD = 1.80) exhibited marginally higher interests in knowing more about the target persons than did those under the excessive covering (*M* = 3.24, SD = 1.44; *t*(24) = 1.79, *p* = 0.08). Also, the analysis of the perception of beautifulness showed that participants under the moderate covering (*M* = 4.76, SD = 1.27) perceived the targets as more beautiful than did those under the excessive covering (*M* = 4.08, SD = 0.49; *t*(24) = 2.59, *p* < 0.05). Last, the analysis on the first impression of coldness (vs. warmth) indicated that participants in the excessive covering (*M* = 6.24, SD = 1.42) perceived more coldness than did those in the moderate covering (*M* = 4.84, SD = 1.21; *t*(24) = −4.67, *p* < 0.001), indicating that excessive covering hindered positive person perceptions.

**Figure 3:**
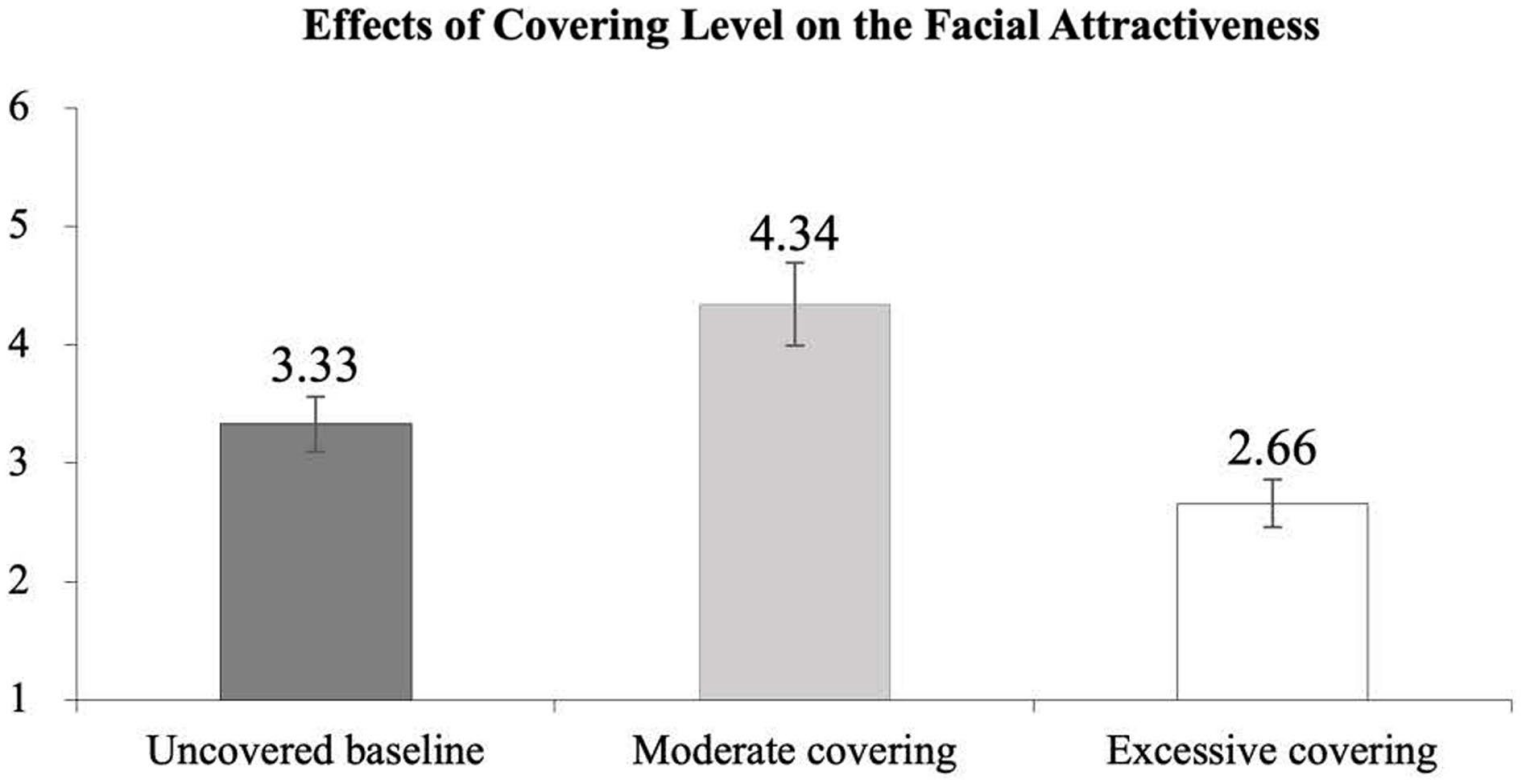
Facial attractiveness and the level of covering. The error bars show ± one standard error calculated from within participant variance.

Next, the analysis of the facial attractiveness index in each of the three covering levels was performed to test the curvilinear effect of wearing a mask. As shown in Figure 3, the analysis of post-hoc comparisons further indicated that the perceived facial attractiveness index was the highest in the moderate covering (*M* = 4.34), followed by in the averaged uncovered baselines (*M* = 3.33) and the excessive covering condition (*M* = 2.66; *F*(1, 24) = 5.78, *p* < 0.05). The analysis of post-hoc comparisons further confirmed that all the pairwise mean difference — the moderate-the excessive covering (*t*(24) = 5.38, *p* < 0.001), the averaged uncovered baselines-the moderate covering (*t*(24) = −2.73, *p* < 0.05), and the averaged uncovered baselines-the excessive covering (*t*(24) = 2.40, *p* < 0.05) — were found statistically different. Thus, the results provided the evidence in support of the curvilinear relationship of the mask covering effect as hypothesized.

### 3.2. The analysis of eye-tracking data

To uncover a psycho-physiological process under which such mask fishing effects occurred, we conducted the analysis of the visual data gathered through the eye-tracker. First, we set up the areas of interests (AOIs) in the stimulus material. Depending on the covering level, the different AOIs were set as cue areas from which inferences about the target persons’ appearance would be made. Specifically, the AOI was set to the forehead and eyes at the top of the mask in the moderate covering condition as the gaze was expected to be mainly directed to those areas when evaluating the masked targets. In contrast, the AOI was set to the eye area between the mask and the bucket hat in the excessive covering condition as the gaze was expected to be limited to the areas around the eyes. The remaining parts, such as the target’s body and background, were neglected and thus presented identically in both conditions.

**Figure 4:**
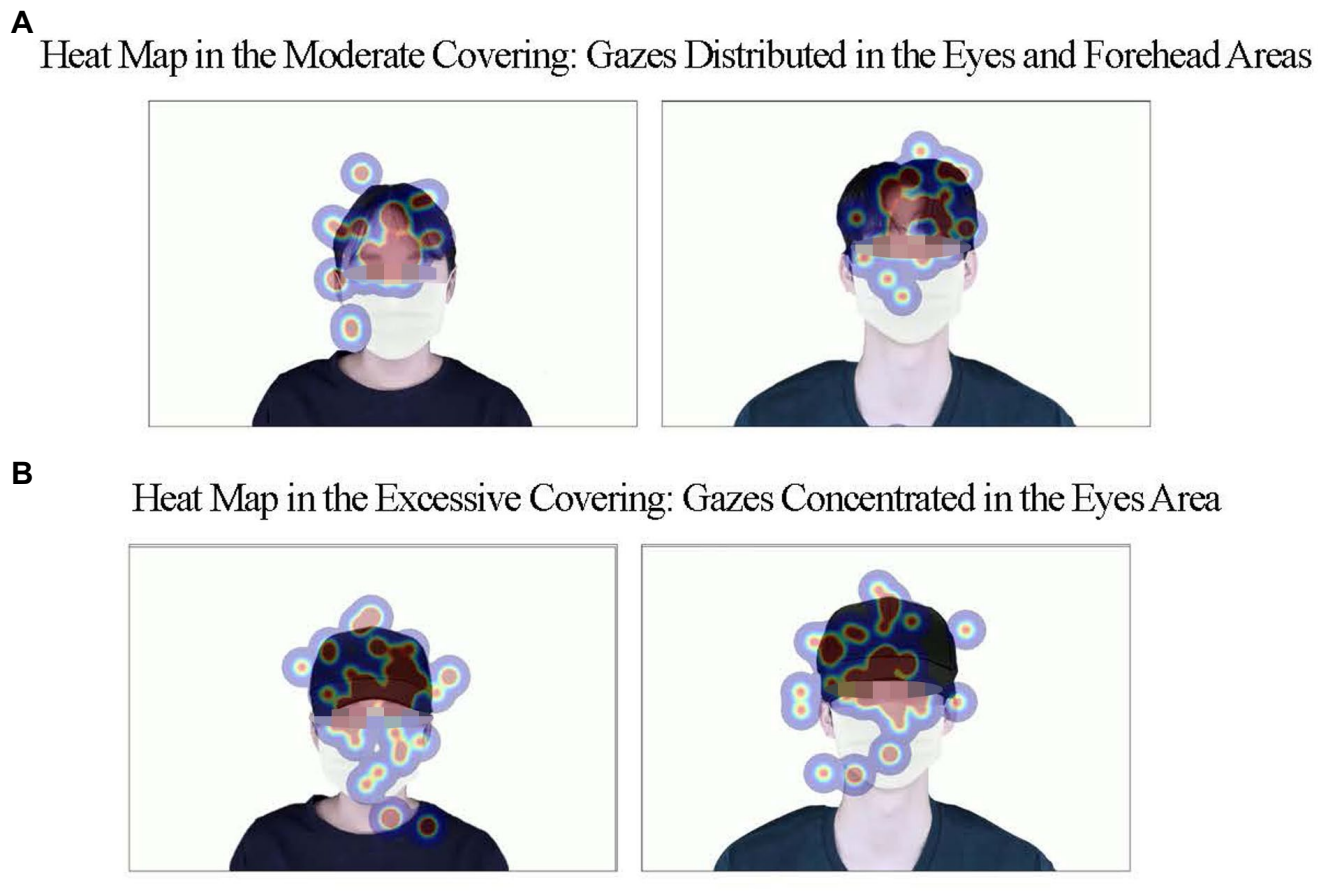
Heat map of fixation with different covering levels (low (in blue), high (in red)). Facial images reproduced with permission from AI-Hub (https://www.aihub.or.kr), operated by the National Information Society Agency in Korea.

Next, we employed the measure of “gaze density” to utilize the previously described cue areas for additional analysis. Given that the fixation and revisit values measured through the eye-tracker could vary according to the size of the AOIs, we set the size of the AOIs differently depending between the moderate and the excessive covering conditions, and then calculated “value per area” rather than a single value for the purpose of standardization of the values. As shown in Table 2, the analysis of the fixation score first indicated that the fixation scores per unit area were significantly different between the moderate covering (*M* = 522.66, SD = 210.28) and the excessive covering condition (*M* = 681.75, SD = 423.08; *t*(24) = −2.13, *p* < 0.05), suggesting that the viewers under the excessive covering had no choice but to focus on the target persons’ eyes most of the time due to the eyes being the only cue area when evaluating their facial attractiveness. In contrast, the viewers under the moderate covering were able to evenly distribute the gaze to the forehead and eyes areas because the cue areas were relatively wide relative to the those under the excessive covering. Therefore, participants under the moderate covering exhibited a relatively low fixation per unit area than did those under the excessive covering. The heat map shown in Figure 4 provides a pictorial description of the fixation per unit outcomes as explained above. Second, the analysis of the revisit score also revealed that the number of revisit in the excessive covering (*M* = 560.00, SD = 464.27) was significantly higher than the number in the moderate covering (*M* = 322.02, SD = 188.67; *t*(24) = −2.91, *p* < 0.01), demonstrating that the gaze revisited more times in the process of inferring and evaluating the facial attractiveness of the targets in the excessive covering than in the moderate covering condition due to the cues being limited to the eyes area.

**Table 2:** Eye-tracking data analysis: means and standard deviations (in parentheses).

Density of AOI	Moderate covering (cue: eyes, forehead) (A)	Excessive covering (cue: eyes) (B)	*t*-Statistic (two-tailed) (A)–(B)
Fixation	522.66 (210.28)	681.75 (423.08)	*t*(24) = −2.13*
Revisit	322.02 (188.67)	560.00 (464.27)	*t*(24) = −2.91**

†*p* < 0.10, *p < 0.05, **p < 0.01, ***p < 0.001.

## 4. Discussion

In this research, we have successfully identified both the positive impact of wearing a mask on the perception of facial attractiveness and the psycho-physiological underlying mechanism of such covering effect through the eye-tracking experiment. The findings provide empirical evidence in support of our hypotheses that when it comes to forming the first impression of other people, individuals are likely to pay their attention to the areas unmasked and to get more curious about those people and the areas masked as well, thereby leading to more positive perceptions of facial attractiveness and beautifulness, but that covering too much of the face does harm rather than good to the perceptions of facial attractiveness because only limited inferences are drawn from the cues available in the small areas unmasked. The findings thus confirms that there is a curvilinear relationship between the amount of the facial area covered and the favorability of facial attractiveness perceptions.

Our work makes meaningful and timely theoretical and practical contributions to the discussion of whether and how wearing a mask can influence an individual’s perception of facial attractiveness particularly at the time when wearing a mask is gradually changing from mandatory to optional worldwide. First, the current research not only documents the additional findings congenial with the previous studies with respect to the positive covering effect (Lin and Zhou, 2021; Parada-Fernández et al., 2022; Dudarev et al., 2022b), but it extends the scope of the extant research further by identifying the curvilinear relationship between the masked areas and the facial attractiveness. In a related vein, the current research also offers much broader implications about the utilization of visual cues and the role of curiosity. As Hies and Lewis (2022) explain, a mask relocates people’s attention to the areas unmasked such as the eyes and forehead, and at the same time the brain overestimates the whole while filling in the uncaptured parts of the face, leading to positive perceptions of facial attractiveness. By analyzing the eye gaze movement data between the masked and the unmasked areas, our eye-tracking experiment provides further insights into the psycho-physiological mechanism under which curiosity stimulates further processing of information with regard to target objects. On the other hand, the current research makes interesting implications to individuals’ health and public safety as we are about to start the era of COVID-19 endemic. That is, public health professionals can still encourage individuals to voluntarily wear a mask by reminding them of the benefits of both the mask-fishing and infectious disease protection according to the findings of the current research.

Despite all the merits, however, the current research also has limitations that offer avenues for further investigation. First, the convenient sample used in the current study is neither representative nor large enough to draw generalizable conclusions across different sample population. Future research thus needs to make sure both the size and the representativeness of the sample are to be adequate to enhance external generalizability. Second, macro-level differences such as socio-cultural differences with respect to practices of wearing a mask (Wada et al., 2012) may exist and account for much variance in the positive or negative impact that wearing a mask has on person perception. Thus, it is more than necessary for future research to take such macro-level differences into consideration for individual and public safety across different societies and cultures. Last, the current research still lacks understanding of what factors mediate the effect of wearing a mask on facial attractiveness although the analysis of the gaze movement between the areas of interests was performed. Further research thus seems warranted in this regard as we are still waiting for an answer to the question of whether it is the intensity of gaze movement, induced curiosity, or another factor that mediates the effect of wearing a mask on person perception.

## Data availability statement

The raw data supporting the conclusions of this article will be made available by the authors, without undue reservation.

## Ethics statement

Ethical review and approval was not required for the study on human participants in accordance with the local legislation and institutional requirements. The patients/participants provided their written informed consent to participate in this study.

## Author contributions

N-HJ and D-HP contributed to conception and design of the study. J-CY organized the database. JL and N-HJ performed the statistical analysis under the guidance of S-BP. N-HJ wrote the first draft of the manuscript. JL, J-CY, D-HP, and S-BP wrote sections of the manuscript. All authors contributed to manuscript revision, read, and approved the submitted version.

## Funding

This work was supported by the Ministry of Education of the Republic of Korea and the National Research Foundation of Korea (NRF-2020S1A5A2A01040055).

## Conflict of interest

The authors declare that the research was conducted in the absence of any commercial or financial relationships that could be construed as a potential conflict of interest.

## Publisher’s note

All claims expressed in this article are solely those of the authors and do not necessarily represent those of their affiliated organizations, or those of the publisher, the editors and the reviewers. Any product that may be evaluated in this article, or claim that may be made by its manufacturer, is not guaranteed or endorsed by the publisher.
